# Gonorrhœal Salpingitis in Children

**Published:** 1904-10-15

**Authors:** 


### GONORRHŒAL SALPINGITIS IN CHILDREN.

That gonorrhœa not infrequently occurs in young children has been a recognised fact for many years, and indeed so prevalent has the disease become in some quarters—for example in the orphanages of New York—that drastic measures have been found necessary to stop the spread of the complaint. As a rule the condition is evidenced chiefly by vulvitis, or, in boys, by urethritis, but as might be expected among the large mass of material for analysis most of the gonorrhœal complications met with in adults are found to occur also in children. Thus, for example, there have been reported instances in plenty of gonorrhœal arthritis, peritonitis, and even pyæmia affecting patients of tender age. It is rather matter for wonder, therefore, that gonorrhœal salpingitis has not been more frequently observed in such cases. Mr. L. A. Bidwell and Dr. George Carpenter both report cases which have been under their care with this complication. In Mr. Bidwell's case the child, who was six years old, was submitted to laparotomy for symptoms of acute abdominal trouble supervening on gonorrhœal vulvitis of some weeks' duration. Double pyo-salpinx was found, and both tubes were successfully removed, the ovaries being left for fear that constitutional after-effects would follow their removal. The vaginal discharge continued after the operation, and only ceased after the uterus had been curetted. Dr. Carpenter's case was that of a child of three and a half years, who had suffered from a vaginal discharge for six weeks and at the time of the first examination had pain in the lower part of the abdomen accompanied by frequency of micturition. Bimanual examination by the rectum showed that the uterine appendages were involved. Subsequently the swellings diminished and the child recovered. The cases are of interest, for they call attention to an occurrence which is possibly more frequent than is at present realised and which must at all events be taken into consideration in dealing with abdominal and pelvic disorders of an acute nature in children.

[1] Brit. Jour. Chil. Dis., Oct. 1904.

